# Prolonged Anesthesia Effects of Locally Administered Ropivacaine via Electrospun Poly(caprolactone) Fibrous Membranes

**DOI:** 10.3390/membranes13110861

**Published:** 2023-10-27

**Authors:** Li Wang, Jiaming Chen, Zicen Li, Fei Guo

**Affiliations:** 1 Department of Anaesthesiology, Central Hospital of Dalian University of Technology, No. 826 Xinan Road, Dalian 116033, China; shelly19850124@163.com (L.W.); lizicen3602@gmail.com (Z.L.); 2 School of Energy and Power Engineering, Dalian University of Technology, No. 2 Linggong Road, Dalian 116024, China; cjm00000@mail.dlut.edu.cn; 3 Graduate School, Dalian Medical University, No. 9 West Section Lvshun South Road, Dalian 116044, China

**Keywords:** prolonged anesthesia, electrospinning, drug-loaded fibrous membrane, Ropivacaine, local administration

## Abstract

Prolonged analgesia is important to safeguard the patient’s comfort and safety during and after surgery in clinical practice. To meet the demand for prolonged analgesia, medical professionals often resort to increasing drug frequency, which may lead to poor patient compliance and serious complications due to drug overdose. Therefore, it is of great interest to develop controlled-release drug delivery systems for local anesthetics, enabling slow and controlled drug release to prolong the analgesic effect and minimize systemic toxicity. In this study, we utilized an electrospinning technique to fabricate nonwoven poly(caprolactone) (PCL) fibrous membranes loaded with Ropivacaine and performed proof-of-principle experiments on both in vitro drug release tests and in vivo animal tests, to further prolong the analgesic effect of Ropivacaine and improve postoperative local pain management and chronic pain treatment. Material characterization and in vitro drug release studies confirmed the feasibility of the Ropivacaine-loaded PCL fibrous membranes for sustained release. The drug loading content and drug loading efficiency of Ropivacaine-loaded fibrous membrane are 8.7 ± 0.3 wt% and 96 ± 3 wt%, respectively. Evaluation in an animal model demonstrated prolonged anesthesia effects along with excellent biocompatibility and stability. At 72 h, the cumulative release accounted for approximately 50% of the drug loading content. This study offers novel approaches and strategies for clinical postoperative pain management and chronic pain treatment, while providing new insights and directions for the design of local anesthetic controlled-release delivery systems.

## 1. Introduction

Effective postoperative pain management is of significant importance during the whole perioperative period. Inadequate postoperative pain management may lead to increased incidence of complications, higher inpatient medical costs, and even a potential for the development of chronic pain (approximately 10%) [1,2]. Currently, the management of both acute and chronic pain still relies on potent opioid medications, including synthetic opioids, e.g., Fentanyl. This is directly associated with the increase in opioid-related mortality rates [3]. It is also one of the primary factors associated with the global opioid misuse crisis [2]. Local anesthetics, as crucial constituents of non-opioid pharmacotherapy, effectively alleviate pain arising from various causes. However, the local anaesthetic systemic toxicity and the short half-life property limit their clinical applications [4,5,6]. To meet clinical analgesic needs, physicians may choose to increase dosing frequency, which potentially leads to poor patient compliance and even serious complications, such as local anesthetic toxicity due to drug overdose. Therefore, it is important to develop a sustained-release drug delivery system for local anesthetics, enabling their gradual and controlled release, with the aim of extending analgesic duration and mitigating systemic toxicity.

Sustained-release delivery systems for local anesthetics have been developed to address the aforementioned issues [7]. In 2011, the first commercialized sustained-release formulation of bupivacaine, EXPAREL^®^, gained FDA approval, asserting the capability to prolong the effect of bupivacaine up to 72 h [8,9]. However, due to the high fluidity of liposomes in EXPAREL^®^, its analgesic efficacy is unstable, which may lead to safety concerns [10,11]. Moreover, an increasing body of evidence suggests that liposomal bupivacaine’s analgesic effectiveness is not superior to non-liposomal bupivacaine during peripheral nerve blocks [12,13,14].

Researchers are continuously exploring avenues for developing advanced sustained-released drug delivery systems of local anesthetics to meet the demands of high biocompatibility, controllable biodegradability, target specificity, and reduced dosing frequency. For example, Y Zhang et al. developed a thermosensitive hydrogel system loaded with levobupivacaine, which exhibited a prolonged analgesic duration seven times longer than regular levobupivacaine [15]. Generally, utilizing various techniques, such as nanotechnology [16], 3D printing technology [17], and electrospinning [18], a variety of formulations, including microparticles [19], nanoparticles [18], liposomes [20], cyclodextrins [21], and hydrogels [15], have been studied for the development of sustained-release systems for local anesthetics. However, some limitations are observed in the existing local anesthetic delivery systems based on diverse release carriers, such as intricate preparation techniques, elevated costs, susceptibility to environmental influences, susceptibility to leakage and burst release, as well as difficulties in tissue adhesion and limited targeting specificity [22,23].

Electrospinning is a fiber production method that applies electric force to draw charged threads of polymer solutions to ultrafine fibers and the corresponding nonwoven fibrous membranes [24]. It is a promising approach for producing various specialized fiber structures. Electrospun fibrous membranes exhibit high specific surface area and porous structure, ensuring substantial drug loading capacity and encapsulation efficiency. Notably, the parameters of these fibrous membranes, including fiber diameter, membrane porosity, and membrane thickness, can be adjusted through manipulation of operating parameters and polymer solution properties [25]. These membranes are potentially suitable as local anesthetic delivery systems with certain release capabilities [25,26].

PCL offers several advantages for biomedical applications, particularly in tissue engineering [27,28], including good biocompatibility, non-immunogenicity, flexibility, and degradation without acidic accumulation. There are studies using electrospun PCL fibrous membranes as controlled-release drug delivery systems in the treatment of cancer, diabetes, and neurological disorders [24,29]. In our study, we explore the utilization of PCL as a carrier for anesthetic agents. Regarding the selection of anesthetic agents, we use Ropivacaine as the experimental compound. Ropivacaine is classified as a long-acting amide local anesthetic agent distinguished by its lower cardiovascular and neurological toxicity and sensory-motor separation properties. This profile has led to wide clinical applications [30].

In this study, we developed PCL fibrous membranes containing Ropivacaine through an electrospinning technique and evaluated their material characteristics, drug release in vitro, prolonged anesthesia effects of locally administered Ropivacaine in vivo, and potential toxicity. The results from the proof-of-principle experiments provide valuable insights into the development of controlled-release drug delivery systems using fibrous membranes as a drug carrier with Ropivacaine as a model drug, showing promising prospects for enhanced pain management in various clinical settings.

## 2. Materials and Methods

### 2.1. Materials

Poly(caprolactone) (M_n_ = 50,000~60,000 g/mol) was purchased from Shanghai Aladdin Bio-Chem Technology Co., Ltd. (Shanghai, China). Ropivacaine (C_17_H_26_N_2_O, 98%, M_w_ = 274.4 g/mol) was obtained from Shanghai Macklin Biochemical Co., Ltd. (Shanghai, China). Dichloromethane (CH_2_Cl_2_, AR) and anhydrous ethanol (CH_3_OH, AR) were procured from Tianjin Kemiou Chemical Reagent Co., Ltd. (Tianjin, China). The solubility of Ropivacaine in water is 10 mg/mL, and it is easily soluble in DCM. Male Sprague Dawley rats were purchased from Liaoning Changsheng Biotechnology Co., Ltd. (Benxi, China). The syringe (Jiangxi Fenglin Medical Appliances Co., Ltd., Fuzhou, China) used for electrospinning was 10 mL. The electrospinning equipment came from Yongkang Leye Technology Development Co., Ltd. (Beijing, China).

### 2.2. Preparation of PCL Fibrous Membranes via Electrospinning

The PCL solution was dissolved in dichloromethane and subjected to stirring at room temperature using a magnetic stirrer (Shanghai Sile Instrument Co., Ltd., Shanghai, China) operated at 500 rpm for 12 h. Following defoaming, the solution was delivered through a 21-G stainless steel needle at a controlled injection rate of 0.02 mL/min. Electrospinning of PCL fibers was carried out within an electrostatic field generated by applying a positive voltage of 12.8 kV and a negative voltage of −3.8 kV. Conditions of controlled temperature (25 ± 1 °C) and humidity (22 ± 7%) were maintained during the process. The receiving collector, positioned at a distance of 20 cm, was set to rotate at a rate of 30 rpm. The electrospinning parameters are presented in Table 1. All membranes were dried at 39 °C for over 12 h to ensure the removal of residual solvent in consideration of the PCL melting point (58–64 °C) and the boiling point of dichloromethane (39.8 °C).

### 2.3. Preparation of Ropivacaine-Loaded PCL Fibrous Membranes via Electrospinning

A composite solution was prepared by dissolving Ropivacaine (2 wt%) and PCL (20 wt%) in dichloromethane (78 wt%). Subsequently, the Ropivacaine-loaded PCL electrospun fibrous membrane (referred to as Ropivacaine-loaded fibrous membrane) was obtained via electrospinning under an electrostatic field with a positive voltage of 11.2 kV, a negative voltage of −3.1 kV, and a collecting distance of 15 cm. The electrospinning parameters and procedures for the Ropivacaine-loaded fibrous membrane were consistent with those for the PCL fibrous membranes without Ropivacaine. The specific operational and material characteristics are listed in Table 1.

### 2.4. Preparation of Ropivacaine-Loaded PCL Dense Membranes

Ropivacaine (2 wt%) and PCL (20 wt%) were mixed and dissolved in dichloromethane (78 wt%), followed by stirring at room temperature for 12 h. The resulting clear solution was poured onto a PTFE plate and evenly spread using a glass slide. After natural drying (approximately 5 min), the membrane that adhered to the plate was peeled off to yield the Ropivacaine-loaded PCL dense membrane (referred to as Ropivacaine-loaded dense membrane, Figure S1 of Supplementary Materials). The membrane exhibited a thickness of about 80 to 120 μm without a porous structure.

### 2.5. Characterizations

The weight of the drug was measured by an electronic balance (BSM220.4, Shanghai Zhuojing Electronic Technology Co., Ltd., Shanghai, China). The accuracy of the balance is 0.1 mg. The image of the pure Ropivacaine crystals was characterized using an optical microscope (D300C, Shanghai Bingyu Optical Instrument Co., Ltd., Shanghai, China). The morphology of the membrane was characterized using a scanning electron microscope (TM4000Plus, Hitachi, Japan). Prior to observation, the membrane surface was sputter-coated with gold for 120 s using an ion sputter coater to enhance conductivity. The porosity was measured using the oil immersion method. The water contact angle was measured by randomly placing 2.5 μL of deionized water on the flat membrane surface. Energy-dispersive X-ray spectroscopy (EDS) analysis of nitrogen elements in the Ropivacaine-loaded fibrous membrane was performed using a high-resolution field emission scanning electron microscope (JSM-7900F, Tokyo, Japan). Additionally, the presence of N-H absorption peaks in the Ropivacaine-loaded fibrous membrane was detected using a Fourier transform infrared spectrometer (IS50, Thermo Nicolet, Madison, WI, USA).

### 2.6. Ropivacaine Release In Vitro Experiments

To assess the drug release profile, 50 mg of Ropivacaine-loaded fibrous membrane was dissolved in 10 mL of methanol. After 30 min of ultrasonication and 30 min of stirring, a 100 μL solution was taken and diluted 100 times. The Ropivacaine concentration was determined using a liquid chromatography–mass spectrometry system (Agilent RRLC/6410B, San Diego, CA, USA). Drug loading content (DLC) and encapsulation efficiency (DLE) were calculated, where DLC represents the mass of the loaded drug divided by the total mass of the membrane, and DLE represents the mass of the encapsulated drug divided by the initial mass of the drug.

For the cumulative release study, 0.2 g of Ropivacaine-loaded fibrous membrane was placed in 10 mL of phosphate buffer solution. Samples were collected at 5 min, 1 h, 3 h, 6 h, 10 h, 24 h, 48 h, and 72 h, each time replenishing the buffer solution. After a 200-fold dilution, the Ropivacaine concentration was measured using the chromatography–mass spectrometry system. The cumulative drug release was calculated using the previously determined drug loading content, thereby generating the percentage release profile of the Ropivacaine-loaded fibrous membrane.

### 2.7. In Vivo Experiments

Fifteen male Sprague Dawley (SD) rats, with weights of 200~250 g, were allowed free access to food and water and were maintained at a temperature of 26 °C and a humidity range of 40–70%. All rats were acclimated for one week prior to the start of the experiments under these conditions. All animal experiments were conducted in compliance with the ethical principles of animal research. Rats were euthanized by overdose of sevoflurane 72 h after surgery.

The rats were randomly divided into three groups, each containing five rats. The test rats were anesthetized through intraperitoneal injection with chloral hydrate (100 mg/mL) at a dosage of 3 mL/kg of body weight. Following the induction of anesthesia, a 2 cm incision was made on the right hind hip, and the muscles were gently separated to expose the sciatic nerve (Figure S2 of Supplementary Materials). In the Ropivacaine-loaded fibrous membrane group, a 10 mg fibrous membrane (1.5 cm × 2 cm, ~1 mg of Ropivacaine) was folded along its shorter axis and placed within the muscle gap along the direction of the sciatic nerve. For the Ropivacaine aqueous injection group, a 10 mg piece of defatted cotton was positioned on the sciatic nerve’s surface in a similar manner to the Ropivacaine-loaded fibrous membrane group. Subsequently, 0.1 mL of Ropivacaine injection solution (10 mg/mL) was evenly administered onto the defatted cotton. A 10 mg Ropivacaine-loaded dense membrane (1.5 cm × 0.7 cm) was placed on the sciatic nerve’s surface with the same method. Neurobehavioral evaluation was blinded to the rats’ groupings.

### 2.8. Anesthesia Effects Evaluation

To evaluate the sensory blockade effect, the Paw Withdrawal Thermal Latency (PWTL) of rats in response to thermal stimuli was measured using a hot plate test. Prior to testing, rats were allowed to acclimate to the environment for 30 min. During the hot plate test, rats were placed on a hot plate with a temperature of 50 °C. An external thermocouple was connected to the testing area of the hot plate to continuously monitor and maintain its temperature stability. The maximum allowable PWTL time was set at 25 s. If a rat did not exhibit escape behaviors (such as paw withdrawal or licking) before this maximum time, it was immediately removed from the hot plate. Each rat underwent the hot plate test for three consecutive days before surgery to establish baseline values for their thermal sensitivity. The average PWTL was calculated by conducting three repeated tests at the planned time points (2, 4, 6, 12, 24, 48, 72 h, post-surgery), with a 10 min interval between each test.

The rats were evaluated for motor function using a four-point scale to assess the effect of the motor blockade. 1 = No motor block. 2 = Dorsiflexion disorder and failure to fully splay the toes when lifting the rat’s tail. 3 = Plantarflexion disorder and complete failure to splay the toes when lifting the rat’s tail. 4 = Complete loss of dorsiflexion and plantarflexion accompanied by gait disorders (Figure S3 of Supplementary Materials).

### 2.9. Local Inflammatory Response and Wound Healing Assessment

The wound healing status of the rats was assessed at 72 h post-surgery. After humane euthanasia of the rats using excess isoflurane, skeletal muscle tissue and the sciatic nerve in close proximity to the implants were retrieved. Following fixation in 4% paraformaldehyde, the specimens were paraffin-embedded, sectioned into 3–4 μm thick slices using a microtome (Leica CM1950, Wetzlar, Germany), stained with hematoxylin and eosin, and sealed with neutral mounting medium. Optical microscopy was employed for observation and imaging. The baseline body weight of each rat before the surgery and their weight 72 h after surgery were recorded and compared to evaluate the systemic impact of different implanted materials on the rats (Figure S4 of Supplementary Materials).

### 2.10. Statistical Analysis

Statistical analysis was performed using GraphPad Prism 8.0.2 software (GraphPad, San Diego, CA, USA). The measured data in the experiment, such as the PWTL, were presented as mean ± standard error (SE). Differences between groups were analyzed using one-way analysis of variance (ANOVA) with repeated measures and Tukey’s multiple comparisons test.

## 3. Results and Discussion

### 3.1. Microstructures and Ropivacaine Integration

**Figure 1:**
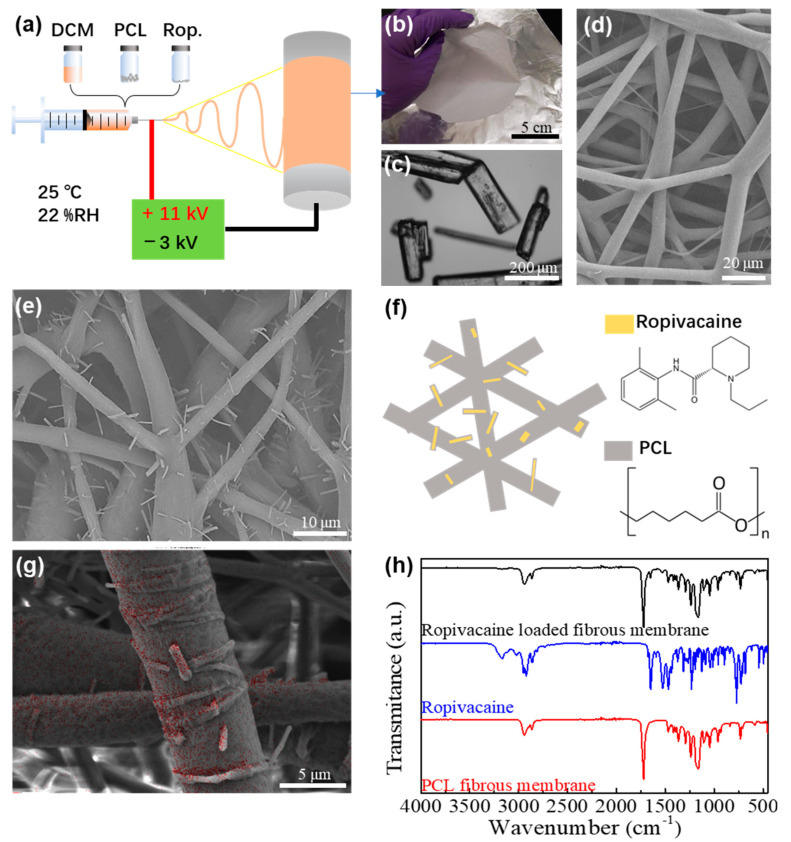
Characterization of Ropivacaine-loaded PCL fibrous membrane using electrospinning. (**a**) Schematic diagram of the electrospinning process with various solutions as the fiber precursors. (**b**) Photographs of the electrospun fibrous membrane collected on the aluminum foil. (**c**) Optical microscope image of the pure Ropivacaine crystals. (**d**) SEM image showing the morphology of the electrospun PCL fibrous membrane with nonwoven, smooth fibers. (**e**) SEM image showing the morphology of the electrospun Ropivacaine-loaded PCL fibrous membrane with branched structure of nonwoven fibers. (**f**) Schematic illustration of the structure of Ropivacaine-loaded PCL fibrous membrane. (**g**) EDS analysis of the Ropivacaine-loaded fibrous membrane. The red dots indicate the presence of nitrogen elements. (**h**) FTIR analysis of the Ropivacaine-loaded fibrous membrane.

The preparation of the Ropivacaine-loaded fibrous membrane involves the introduction of Ropivacaine crystals (shown as rod-like structures in Figure 1c) into a PCL DCM solution. In the electrospinning process, this solution was pulled into micro fibers, which were collected on a rotating drum and formed a nonwoven fibrous membrane. The membrane can be peeled off from the drum as a free-standing Ropivacaine-loaded fibrous membrane. It can be observed from the SEM images that the electrospun PCL fibers without Ropivacaine are smooth on the surface (as shown in Figure 1d), while the fibers with Ropivacaine present needle-like structures on the fiber surface (as shown in Figure 1e). Due to the pre-mixing of Ropivacaine with PCL in dichloromethane prior to electrospinning, the rapid evaporation of the solvent during electrospinning leads to Ropivacaine forming needle-like structures closely integrated with the PCL fibers. Figure 1f illustrates the needle-like Ropivacaine structures anchored onto the PCL fibers. These Ropivacaine needle-like structures on the fibers closely resemble the original crystal morphology of the Ropivacaine powder, but due to the low Ropivacaine content and the rapid solvent evaporation during spinning, the structures are finer and more needle-like in appearance.

Within this system, nitrogen elements are exclusively present in Ropivacaine, whereas PCL lacks nitrogen elements. EDS analysis serves to ascertain the presence of Ropivacaine in the fibrous membranes (Figure 1g), accompanied by an examination of N-H bond absorption peaks using FTIR (Figure 1h). These analyses collectively confirm the successful fabrication of the Ropivacaine-loaded fibrous membrane. The presence of Ropivacaine within the fiber membrane is evident, and the needle-like structures observed on the coarse fiber strands correspond to Ropivacaine. The measurement of nitrogen content is influenced by multiple factors and may not be precise; however, it remains suitable for qualitative analysis.

### 3.2. Ropivacaine Release from PCL Carriers In Vitro

**Figure 2:**
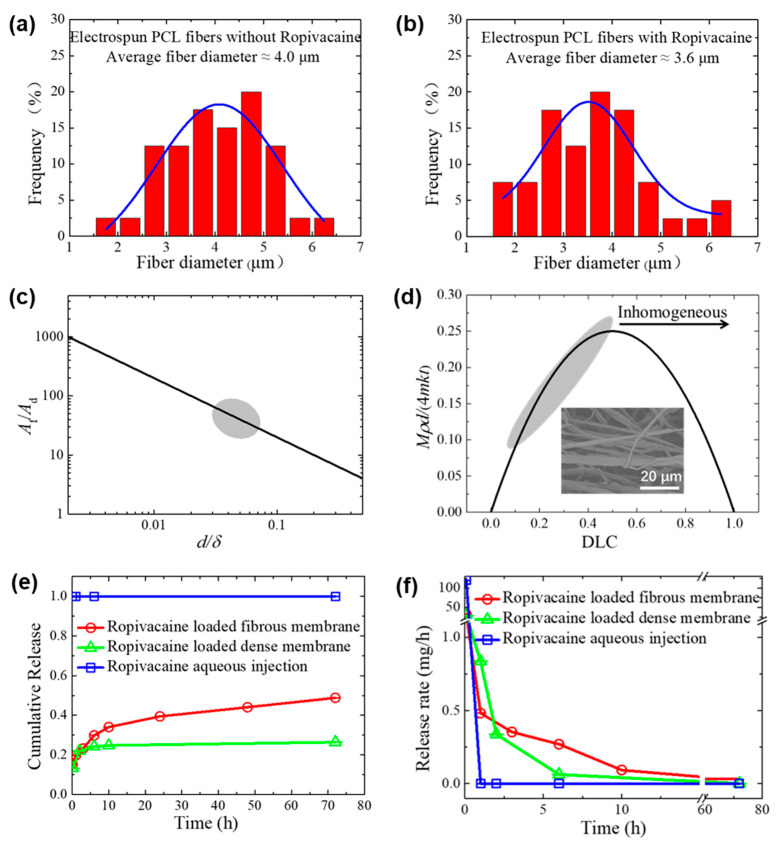
In vitro release of Ropivacaine from PCL carriers. (**a**) Statistical distribution of fiber diameter of electrospun PCL fibers. (**b**) Statistical distribution of fiber diameter of electrospun PCL fibers with Ropivacaine. (**c**) Comparison of the relationship and range between the surface area and fiber diameter of fibrous membranes in contrast to dense membranes. The variable *d*/*δ* indicates the ratio of fiber diameter to membrane thickness, while *A*_f_/*A*_d_ indicates the ratio of fiber surface area to membrane surface area. (**d**) Theoretical relation between drug loading capacity (DLC) and drug release rate. (**e**) Cumulative release amount and (**f**) release rate of Ropivacaine over time with various drug-carrying configurations.

Figure 2a,b present a statistical distribution of fiber diameters for the electrospun PCL fibrous membrane with Ropivacaine and without Ropivacaine, with an average fiber diameter of 4 μm and 3.6 μm, respectively. The introduction of Ropivacaine into the fibers slightly decreases the fiber diameter due to the reduction of solution viscosity during the electrospinning process. For a dense membrane, the surface area increases as the membrane thickness decreases. For a fibrous membrane, its surface area further enlarges as the fiber diameter decreases. The fibrous membrane exhibits a significantly larger surface area compared to the dense membrane, often reaching approximately 10–100 folds, as illustrated in Figure 2c.

(1) $$\frac{A_{f}}{A_{d}}=\frac{2\delta }{d}$$

where *A_f_* and *A_d_* represent the surface areas of the fibrous membrane and the dense membrane, respectively; *d* denotes the fiber diameter of the fibrous membrane; and *δ* denotes the membrane thickness.

The drug relate rate can be evaluated by the following equation:

(2) $$\frac{M}{t}=kAc$$

where *M* indicates the amount of drug released. *t* denotes time; *k* denotes an empirical coefficient, characterizing the collective influence of diffusion coefficients, membrane attributes, and other parameters under the same conditions aside from surface area *A* and concentration *c*. A comparison of diffusion coefficients for several common substances is discussed in Figure S5 of the Supplementary Materials. The calculated diffusion coefficient for Ropivacaine is approximately 4 × 10^−10^ m/s^2^, which is smaller than that of sodium and potassium ions and similar to glucose. By incorporating the derivation of the surface area in Equation (1) and substituting the drug loading content (DLC) for drug concentration, the following expression can be obtained:

(3) $$\frac{M}{t}\sim \frac{4m}{\rho d}k\left(1-\mathrm{DLC}\right)\mathrm{DLC}$$

where *m* and *ρ* denote the mass and density, respectively, of the fibrous membrane. This perspective also underscores that approaching a drug loading close to 1 not only affects the rate during steady release but may also hinder further drug release. In Figure 2d, the alteration in the microstructure of the fibrous membrane with increased DLC also demonstrates that elevating the DLC impacts the formation of Ropivacaine needle-like structures, resulting in partial fiber coverage by the drug and instances of fiber rupture.

The determination of concentrations in solutions using a chromatography–mass spectrometry tandem system yielded the drug loading content (DLC) and drug loading efficiency (DLE) of Ropivacaine-loaded fibrous membrane as 8.7 ± 0.3 wt% and 96 ± 3 wt%, respectively. The minimal drug loss in electrospinning significantly surpasses the requirement for DLE to exceed 80 wt%. For the Ropivacaine-loaded fibrous membrane in vitro diffusion experiments in PBS, substantial release occurred in the initial minutes, followed by gradual and sustained release over time, reaching an equilibrium release rate (Figure 2f). At 72 h, the cumulative release accounted for approximately 50% of the drug loading content (Figure 2e). Conversely, for Ropivacaine aqueous injection, we consider the solution to be homogeneous, leading to direct and complete release. The initial release rate can be deemed infinitely high, subsequently transitioning to no further release (rate considered zero). The data in the graph represent relative results based on initial concentration and time calculations.

The Ropivacaine-loaded dense membrane also exhibited an initial burst release, followed by a rapid decrease in release rate approaching zero. At 72 h, the cumulative release reached around 25% of the drug loading content. We assume that both drug-loaded membranes possess a certain amount of drug not constrained by PCL on their surface, resulting in the initial rapid release. However, the expansive diffusion area during the release of the Ropivacaine-loaded fibrous membrane ensures sufficient drug release and a stable release rate. On the contrary, the lack of voids in the interior of the Ropivacaine-loaded dense membrane due to encapsulation by PCL impedes drug release, leading to considerably higher release resistance compared to the fibrous membrane. The marginal release rate could be attributed to the ongoing degradation of PCL within the dense membrane. Previous studies have indicated that PCL with a molecular weight above 60,000 can degrade over a period of 2 to 3 years, with lower molecular weights leading to shorter degradation times [31]. With enzymatic assistance, PCL degradation can be accelerated to a few months [32], thus supporting the inference of a slow drug release rate within the Ropivacaine-loaded dense membrane.

### 3.3. Prolonged Anesthesia Effects

**Figure 3:**
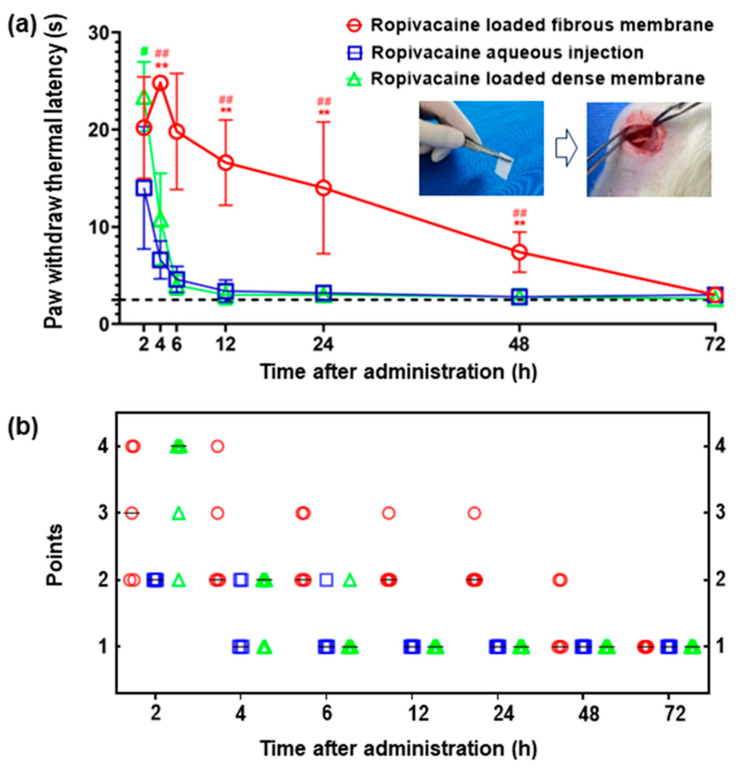
Anesthesia effects of locally administered Ropivacaine in vivo with various drug-carrying configurations. (**a**) The Paw Withdrawal Thermal Latency (PWTL) of rats over time after sciatic nerve blockade by Ropivacaine. The dashed line indicates the baseline of PWTL, which is the mean time of PWTL for all rats before surgery. #: *p* < 0.05; ##: *p* < 0.01 vs. Ropivacaine aqueous injection; **: *p* < 0.01 vs. Ropivacaine-loaded dense membrane. (**b**) Motor block of rats evaluated using a four-point rating scale: 1 = no motor block, 2 = dorsiflexion disorder and failure to fully splay the toes when lifting the rat’s tail, 3 = plantarflexion disorder and complete failure to splay the toes when lifting the rat’s tail, 4 = complete loss of dorsiflexion and plantarflexion accompanied by gait disorders. –: median. n = 5 per group.

Figure 3 presents a comparative analysis of sciatic nerve sensory blockade in rats among the three groups after drug administration. Two hours post-surgery, noticeable extension of the Paw Withdrawal Thermal Latency (PWTL) was observed in all groups, signifying the onset of effect of the three Ropivacaine-containing implants within the rats’ bodies. Rats in the Ropivacaine-loaded fibrous membrane group maintained an extended PWTL within the initial 6 h post-surgery, followed by a gradual reduction until reaching baseline levels by 72 h post-surgery. This trend aligns with the cumulative drug release pattern observed in vitro, with substantial release occurring in the first 6 h followed by gradual and steady release, indicating consistent drug diffusion. In inter-group comparisons, the PWTL for the Ropivacaine-loaded fibrous membrane group was significantly higher than the other two groups at 4, 6, 12, 24, and 48 h post-surgery. Beyond 2 h post-surgery, both Ropivacaine aqueous injection and Ropivacaine-loaded dense membrane groups exhibited swift PWTL reduction, recovering to pre-surgery levels by 12 h post-surgery. This trend mirrored the in vitro drug release results, implying the absence of sustained drug release for these two administration methods. Furthermore, the time to motor function recovery reaching a score of 1 was 48 h for the Ropivacaine-loaded fibrous membrane group, 4 h for the Ropivacaine aqueous injection group, and 6 h for the Ropivacaine-loaded dense membrane group. This indicates that the motor blockade time was longer for the Ropivacaine-loaded dense membrane group compared to the other two groups, confirming the prolonged anesthetic effect of Ropivacaine-loaded fibrous membrane.

The electrospun fibrous membranes exhibit a high surface-area-to-volume ratio and porous structure, ensuring substantial drug loading capacity. This is consistent with our experimental findings, where the drug loading content of Ropivacaine on the PCL electrospun fibrous membrane was measured at about 9 wt%. In the in vitro cumulative drug release profile, a steeper slope is observed within the initial 2.5 h. Our animal experiments indicated that the most potent anesthetic effect was observed at 4 h after administration. This phenomenon suggests that the Ropivacaine-loaded fibrous membrane may experience a burst release of the drug within the initial hours. This can be attributed to the occurrence of drug adsorption on the surface of the high-molecular-weight polymer solution, leading to the presence of unencapsulated drug molecules on the surface of the spun fibers. Additionally, upon exposure to a liquid environment, the liquid infiltrates into the carrier’s pores, causing a partial washout of the drug, thus contributing to the initial burst release phenomenon.

The Ropivacaine-loaded dense membrane, due to its non-porous structure, impedes the release of the encapsulated drug, rendering it incapable of facilitating controlled and sustained drug delivery. The transient anesthetic effect observed with the Ropivacaine-loaded dense membrane group may be attributed to the influence of the portion of Ropivacaine situated on the membrane’s surface. Once these limited drug reserves are exhausted, the Ropivacaine encapsulated within the dense membrane cannot be further released rapidly or sufficiently to exert its anesthetic effect.

### 3.4. Characterization of PCL Fibrous Membranes after Ropivacaine Release In Vivo

**Figure 4:**
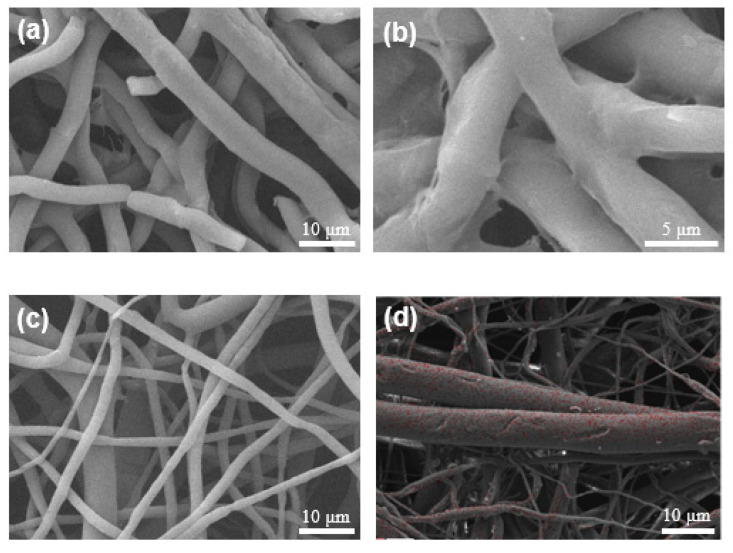
Characterization of Ropivacaine-loaded fibrous membrane after drug release. (**a**) Microscopic view of the retrieved drug-loaded fibrous membrane from rats, revealing fiber fracture due to drug release. (**b**) Microscopic view of the retrieved drug-loaded fibrous membrane from rats, displaying adhesion of rat tissues onto the membrane surface. (**c**) Microstructure of the Ropivacaine-loaded fibrous membrane after release in methanol. (**d**) EDS analysis of the Ropivacaine-loaded fibrous membrane after release in PBS, indicating reduced drug content and residue.

Figure 4 illustrates the microstructural changes of the Ropivacaine-loaded fibrous membrane within rat tissues after drug release and carrier degradation. It is notable that the absence of needle-like structures on the fiber surface indicates the completion of drug release. The PCL fibers display evident fracture patterns (Figure 4a), suggesting observable degradation of PCL within rat tissues. Adhesion between certain fibers and rat tissues is apparent (Figure 4b). Following determination experiments for Drug Loading Content (DLC) and Drug Loading Efficiency (DLE), the Ropivacaine-loaded fibrous membrane was extracted post methanol dissolution and subsequently washed for SEM analysis, as depicted in Figure 4c. The disappearance of the previously seen needle-like structures confirms the attachment of Ropivacaine to the fibers, corroborating the side view perspective. Figure 4d showcases the Energy Dispersive X-ray Spectroscopy (EDS) image of the Ropivacaine-loaded fibrous membrane after extraction from PBS, demonstrating the absence of needle-like structures. The nitrogen element content is measured at a mere 0.33 wt%, suggesting a comprehensive release of Ropivacaine through the PCL carrier within PBS. The presence of certain spots on the surface might be attributed to residue from the dried solution. It is important to note that the drug-loaded fibrous membrane after release was not subjected to any post-release cleaning treatment, distinguishing it from the effects of direct methanol dissolution. Figure S6 of the Supplementary Materials shows the microstructure of the test PCL fibrous membrane during drug release and PCL degradation in rats over time.

### 3.5. Biocompatibility and Safety Assessment of PCL Fiber Membranes

**Figure 5:**
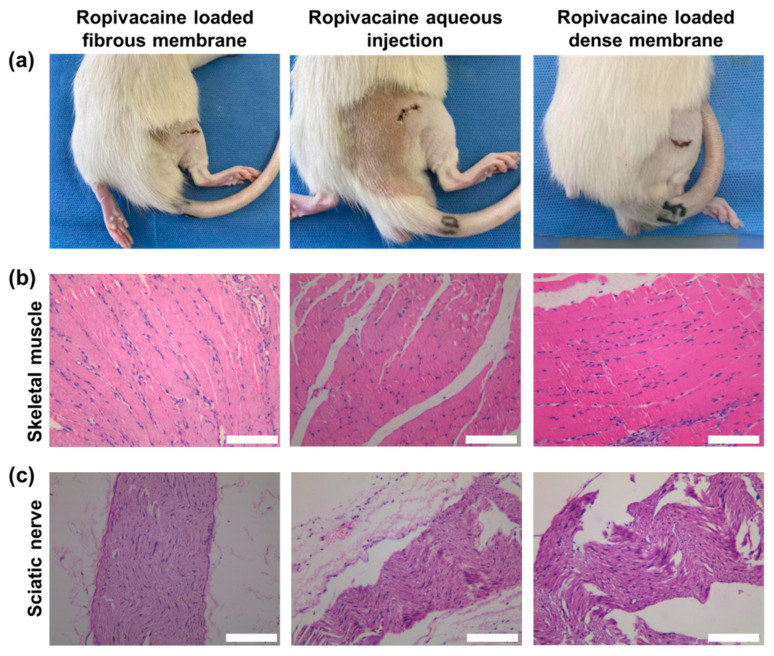
Local inflammatory response and wound healing in rats after surgery involving Ropivacaine delivery with different drug-carrying configurations. (**a**) Photograph of wound healing in rats at 72 h post-surgery. (**b**) Optical microscopy image of HE-stained skeletal muscle tissue around the implanted material in rats. (**c**) Optical microscopy image of HE-stained sciatic nerve around the implanted material in rats. The scale bars indicate 100 μm in the images.

The properties of PCL, such as its non-immunogenicity, minimal acid buildup during degradation, excellent biocompatibility, flexibility, and high mechanical strength, have been reported in previous studies [24]. These findings align with our experimental results. As depicted in Figure 5a, postoperative wound healing was favorable in all three groups of rats, with no significant signs of redness, swelling, or exudation observed around the incision sites. Histological analysis of skeletal muscle tissue surrounding the implants at 72 h post-surgery, as shown in Figure 5b, revealed well-organized muscle fiber arrangement without evident muscle cell necrosis or inflammatory cell infiltration. The HE staining image of the adjacent sciatic nerve in Figure 5c demonstrated dense and structurally normal nerve fiber arrangement, with no pathological changes, such as inflammatory cell infiltration or nerve demyelination. The body weight of rats that received different implants did not exhibit significant changes compared to their baseline weight at 72 h post-implantation, indicating the absence of systemic toxicity caused by the PCL fibrous membrane implantation. Our experimental findings are consistent with previous research, supporting the high biocompatibility of PCL Ropivacaine-loaded fibrous membranes for safe application in biological systems.

## 4. Conclusions

In this study, we successfully developed poly(caprolactone) (PCL) nonwoven fibrous membranes loaded with Ropivacaine using the electrospinning technique. The fibers have a micron and submicron diameter and carry needle-like Ropivacaine crystals within. The fibrous membrane exhibits a 10–100 fold surface area compared to the dense membrane. The drug loading content (DLC) and drug loading efficiency (DLE) of the Ropivacaine-loaded fibrous membrane were 8.7 ± 0.3 wt% and 96 ± 3 wt%, respectively. The tunable electrospinning parameters determine the drug release rate and pattern, allowing for the loading of a significant amount of medication. The fibrous membrane can be delivered through surgical incisions and placed near the nerves. As PCL degrades and small gaps exist in the fibers, Ropivacaine is continuously released in the body, providing prolonged anesthesia for more than 48 h, as confirmed by in vitro drug release studies and in vivo animal tests. This study provides valuable insights into the use of fibrous membranes as drug carriers and highlights the advantages of the electrospinning technique and PCL fibers in drug delivery and tissue engineering applications. Further research and development in this area could contribute to improved pain management and patient outcomes.

## Figures and Tables

**Table 1:** Electrospinning processing parameters and the resulting fibrous membrane properties.

Parameters	Unit	Electrospun PCL Fibrous Membrane	Electrospun PCL Fibrous Membrane with Ropivacaine
Solution Composition	wt%	PCL/DCM (20/80)	Ropivacaine/PCL/DCM (2/20/78)
Flow Rate	mL/min	0.02	0.02
Voltage	kV	+12.8/−3.8	+11.2/−3.1
Rotating Speed	rpm	30	30
Receiving Distance	cm	20	15
Porosity	%	80 ± 5	72 ± 6
WCA	°	121 ± 5	125 ± 5
Thickness	μm	70 ± 10	90 ± 10
Fiber Diameter	μm	4	3.6

## Data Availability

Data is contained within the article or supplementary material.

## Supplementary Materials

The following supporting information can be downloaded at: https://www.mdpi.com/article/10.3390/membranes13110861/s1.
