# Supplementary material for: Prolonged Anesthesia Effects of Locally Administered Ropivacaine via Electrospun Poly(caprolactone) Fibrous Membranes
Source: Membranes (Basel). 2023 Oct 27;13(11):861. doi: 10.3390/membranes13110861 (PMC10672809; doi:10.3390/membranes13110861)
Supplement: Supplementary file 1 [file membranes-13-00861-s001.zip › membranes-2640098-supplementary.pdf]

Supplementary Information (SI) for

Prolonged Anesthesia Effects of Locally Administered Ropivacaine via Electrospun PCL fibrous Membranes

Index for this document:

1. The morphology of the Ropivacaine loaded PCL dense membrane

2. Surgical procedures for in vivo experiments

3. Motor block of rats evaluated using a four-point rating scale

4. The weight variation of rats

5. Drug loading capacity evaluation and diffusion coefficient

6. Degradation of PCL fibrous membrane in rats

1. The morphology of the Ropivacaine loaded PCL dense membrane

The Ropivacaine-loaded PCL dense membrane doesn't have porous structure. Because the thickness of the dense membrane coating is not uniform enough, the surface microstructure is not uniform (as shown in Fig.S1a). Some areas on the surface have Ropivacaine exposed on the surface (as shown in Fig.S1b), while some areas completely seal Ropivacaine inside the dense membrane.

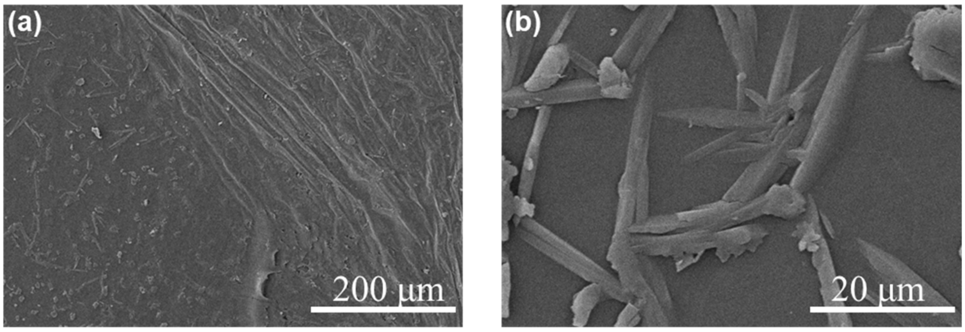

Figure S1. The morphology of the Ropivacaine loaded dense membrane. (a) The larger view. (b) Partial enlarged view.

2. Surgical procedures for in vivo experiments

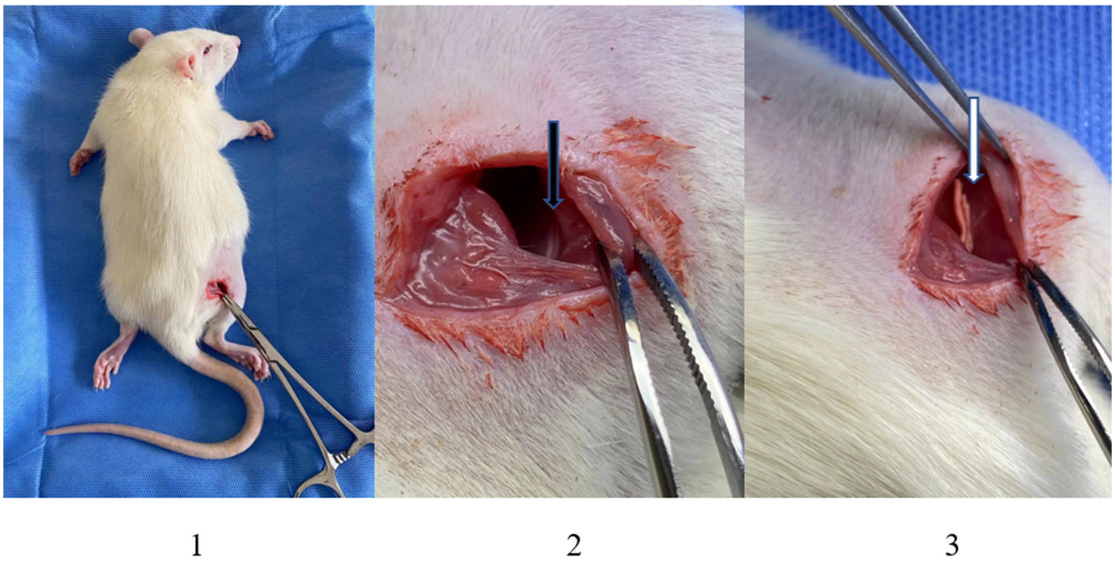

Figure S2. Surgical procedures for in vivo experiments**.** 1: After anesthetizing the rat, make a skin incision in the hip area, bluntly dissect the muscles, and carefully isolate the sciatic nerve. 2: The black arrow indicates the location of the sciatic nerve. 3: Fold the membrane and place it along the longitudinal axis on the surface of the sciatic nerve. The black arrow indicates the location of the PCL fibrous membrane.

3. Motor block of rats evaluated using a four-point rating scale

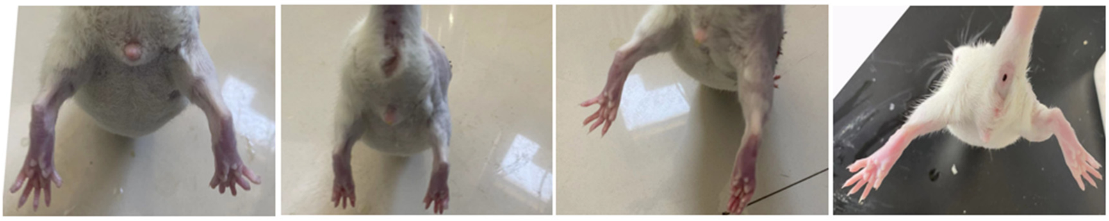

Figure S3 Motor block of rats evaluated using a four-point rating scale. 1 = no motor block, 2 = dorsiflexion disorder and failure to fully splay the toes when lifting the rat's tail, 3 = plantarflexion disorder and complete failure to splay the toes when lifting the rat's tail, 4 = complete loss of dorsiflexion and plantarflexion accompanied by gait disorders

4. The weight variation of rats

The baseline body weight of rats in each group before the surgery and their weight 72 hours after surgery were recorded and compared to evaluate the systemic impact of different implanted materials on the rats. There was no significant difference in the body weight of the rats after implantation of various drug-loaded configurations.

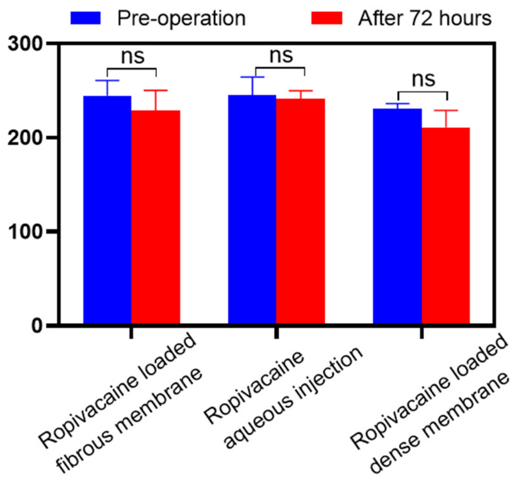

Figure S4. Body weight change of rats 72 hours after implantation of different drug-loading configurations compared to pre-operation.

5. Drug loading capacity evaluation and diffusion coefficient

We can also roughly calculate DLC based on the microscopic image of the Ropivacaine loaded fibrous membrane. We count the number of Ropivacaine drugs on a certain length of PCL fibers in the image, calculate the drug volume and mass based on the shape of the loaded Ropivacaine, and then calculate the mass of this fiber segment.

$$DLC = \frac{n d_1^2 l_1 \rho_1}{d_2^2 l_2 \rho_2 + n d_1^2 l_1 \rho_1} \quad (1)$$

Where d_1 and d_2 are the sizes of drug molecules and fibers, respectively; ρ_1 and ρ_2

represents drug and fiber density, respectively. The estimated DLC is about 2.6%, which is much lower than the calculated concentration of Ropivacaine in the solution measured by the chromatography-mass spectrometer described in the Section 2.6. It indicates that there may be a large amount of Ropivacaine dispersed in the membrane pores rather than fully inserted on the fiber surface.

We estimated the diffusion coefficient of Ropivacaine in water based on the Stokes-Einstein formula:

$$D = \frac{k_B T}{6\pi\mu R_0} \tag{2}$$

where k_B is the Boltzmann constant; T is the solution temperature, K; μ is the viscosity of the solution, Pa · s; R_0 is the radius of the solute molecule, m. Fig.S5 is a comparison between the calculated diffusion coefficients of Ropivacaine and the diffusion coefficients of several common substances. The calculated diffusion coefficient for Ropivacaine is approximately 4×10^{-10} m/s^2, smaller than that of sodium and potassium ions, similar to glucose and much greater than macromolecular organic compounds such as hemoglobin.

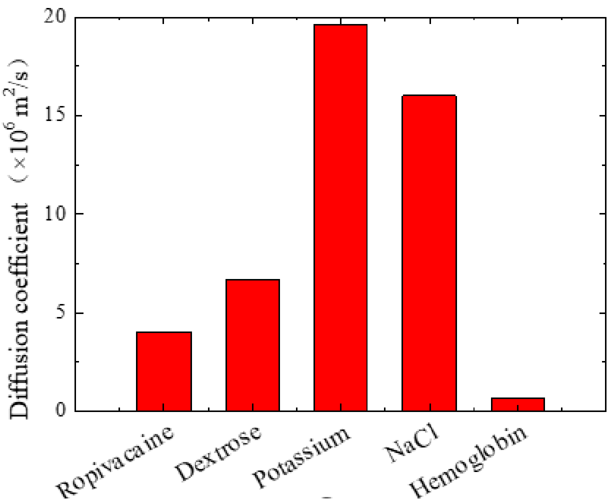

Figure S5. Diffusion coefficients of common substances in water.

6. Degradation of PCL fibrous membrane in rats

Fig S6 shows the microstructure of test PCL fibrous membrane during drug release and PCL degradation in rats over time. It can be seen that there is no needle-like structures on the fiber surface, and the drug has been released. As time increases, the fiber breakage effect significantly increases, and the carrier (PCL fiber) has undergone observable degradation behavior in rats.

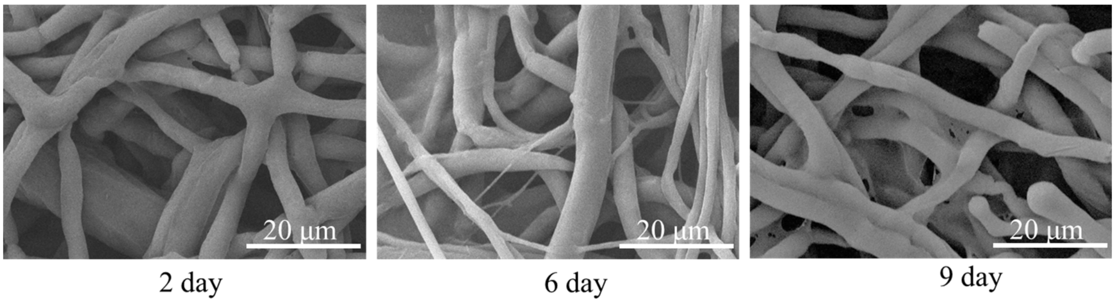

Figure S6. The degradation of Ropivacaine loaded fibrous membrane in rats
